# The Education of the Dentist

**Published:** 1874-09

**Authors:** S. P. Hildreth

**Affiliations:** Retiring President


### THE EDUCATION OF THE DENTIST.

BY S. P. HILDRETH, M. D., D. D. S., RETIRING PRESIDENT.

*A valedictory address read before the Northern Ohio Dental Association, June 10th 1874.*

*Gentlemen of the Northern Ohio Dental Association:*—I have taken as a theme for a few remarks at this time, "The Education of the Dentist."

To an educated man, no apology will be necessary for the selection of this subject, for such an one already feels its importance; and to an ignorant one none should be made for urging the necessity of a full and complete education. I speak of the education of the dentist, as distinguished from a "Dental Education." The latter is the subject of frequent remarks in conventions, and of numerous essays in our periodical literature. By the former term I wish to embrace a wider field, and take in a more comprehensive view than is understood by a mere technical education.

It is true that no man can be a dentist without a thorough Dental Education, and yet a mere technical training in any art or profession does not, by any means, constitute an educated man; and it is because there are so many ignorant, illiterate men engaged in the practice of our profession that I have chosen to present my theme in its persent form.

A calling that is worthy to be dignified with the title of a profession, deserves, and requires more than a mere training in the mechanical manipulations of the art. One who knows no more, and aspires to no more than this, is no better than a cobbler, or a tinker; he is, in fact, a mere animated machine, and might be a legitimate subject for a patent; only such machines are so common that a claim could not be based upon any thing they possess in the way of originality.

By the term, the Education of the Dentist, then, I embrace that higher broader trainings, both of the body and the mind, which should always precede that more special training which qualifies one to practice a particular calling. I use the word education in its primitive and fullest sense. I would have it literally a "leading out" of all the powers and faculties, so that the individual might start in the race of life a complete and perfect man "*Sano mens in corpore sano*," and then, when so prepared for a start in the great struggle of life, I would add a full and thorough training for the special art or profession to be followed.

There can be no great mental strength without a careful and diligent training. As the healthiest child may be sickened and dwarfed by unhealthy and insufficient food, so may the best natural mental gifts be perverted and destroyed by an injudicious training, or by wanton neglect of the means of education. The world gives us no example of an intellectual athlete who has not from the first been snbject to rigid systematic disclipline A man may be a follower, an imitator, without much study, and if he is content to be a lazy shadow, he need not exercise his own powers; but if he would stand forth in all the fullness and strength of a man, he must observe, study, and think.

It is such a training as I have mentioned that alone can fully qualify a man for a thorough dentist, for though our special field of practice is a limited one, there is in connection therewith enough to call forth and engage the best faculties of the ablest minds. We have to deal with the great problem of life from its earliest commencement to its close. We have to question nature as.to some of her most hidden processes, we start from the intangible and uncertain and grope our way to the tangible and the real. Often we have to apply the strictest processes of logic to enable us to trace out cause and sequence. We require a complete knowledge of most branches of natural science. Physical and vital chemistry, botany, climatology, anatomy physiology and materia medica all require to be known that we may intelligently form our conclusions. Without this knowledge, and without the intelligence to apply it, no man is quite prepared to enter upon the study and practice of dentistry. With this preliminary training and knowledge he can work with a hope of distinction and success, for narrow as is our range of practice, there are principles yet undiscovered; there are better modes of practice, and surer methods of treatment which will only be discovered by the men among us who think for themselves.

This educated power is what qualifies a man for an original investigator. The ignorant slave may occasionally blunder upon a jewel, but the educated geologist knows where to look with certainty for the hidden treasures of the mines. The unlearned peasant can see "the glory of the heaven," but only a Galileo or a Newton can trace out the laws that hold the universe in place.

Much has been said and witten upon the right of dentistry to be recognized as a profession. As a branch of the healing art,—and a very important one at that,—there can be no doubt of its intrinsic value to the human race; but as we judge of an occupation by the standing and character of those who follow it; is it any wonder that other professions are slow to to extend to us "the right hand of fellowship?" Look at the mass of practicing dentists and say if their qualifications and and attainments are such as to entitle them to be called members of a learned profession. In a recent conversation with a dentist of some eight or ten years practice (and by the way a licensee of the state board of examiners) he very seriously related what he called a remarkable case of "third indentation." True we have a few "bright and shining lights," men worthy to be called masters of the profession, who are doing all they can to elevate their co-laborers, but by far the larger portion would disgrace a cobler's bench, or a tinker's stool. If we would be recognized as a profession we must first make ourselves worthy, and then absolutely refuse to take any one as a student who has not first laid a sure foundation for success by a liberal education. Do this, and there will be no necessity for laws regulating the practice of dentistry. The empiric and quack will disappear before the magic of intelligence and the profession and the public be happily rid of them forever. Then, and not until then, will dentistry take rank and be recognized as one of the learned professions.

But further than this, though not so gratifying to a man of culture, is the standing that education gives with the general public. If "knowledge is power" then the power to win and hold the favor of many on whom our patronage depends is certainly greatly to be desired; and the public will not always be slow to discern between scientific intelligence and emperical pretention.

And now I come to the selfish reasons for a higher education. Our calling is a laborious one, wearing both upon the mind and the body. The brain wearies with the constant study of decay and its causes. The eye gets tired of viewing unsightly cavities; and the skilled hand would rest from its efforts to arrest decay and restore the lost organs. Let us turn, then, trom these disagreeable sights and seek relaxation and instruction at the same time. The natural sciences afford a never failing source of enjoyment wherever we may be, and are at the same time the best educators. Botany, geology, mineralogy, chemistry and natural philosophy may be studied everywhere, opening the secrets of material nature, shining with recent and brilliant discoveries, and offering the the richest rewards to their cultivators; and they have the highest influence, disciplining the mind and cultivating the taste. They reveal to the soul what has been aptly termed "the poetry of nature and the foundation of art;" and though a dentist need not of necessity be a poet, he should be so much of an artist that his work may rival that of nature in its most perfect forms. In all these pursuits the dentist should act upon the doctrine of the Grecian poet:

"I seek what's to be sought,
I learn what's to be taught,
I beg the rest of Heaven."

Finally I would urge the necessity of a more thorough education as a foundation for that highest of all characters, the ability, and the will, to be a man. To be a man implies much that is not apparent at a superficial view. There must be such physical and mental development and symmetry that all the powers are in perfect harmony. A stimulated, abnormal growth will as surely produce a monster as will a depraved and deficient nutrition. The balance must be kept, so that no portion of the man, neither the body, the mind, nor the religious element may have preponderance the one over the other. While one seeks that which is for our own highest good we must remember that we are but one of the great moving throng of humanity, all of whom have similar objects and desires with ourselves. We seek our own highest good, but we should not forget the welfare of our fellow men. And so, with a generous liberality which makes us tolerant of the peculiarities and opinions of our fellow men, we may press forward in the highway of knowledge, knowing full well:

"He can't be wrong, whose life is in the right."

